# A method for brain tumor detection and localization by means of deep learning and reliable prediction explainability

**DOI:** 10.1371/journal.pone.0361135

**Published:** 2026-10-29

**Authors:** Marcello Di Giammarco, Antonella Santone, Mario Cesarelli, Fabio Martinelli, Francesco Mercaldo

**Affiliations:** 1 Department for High Performance Computing and Networking (ICAR), National Research Council of Italy (CNR), Rende, Italy; 2 Department of Medicine and Health Sciences “Vincenzo Tiberio”, University of Molise, Campobasso, Italy; 3 Department of Engineering, University of Sannio, Benevento, Italy; Bayer Crop Science United States: Bayer CropScience LP, UNITED STATES OF AMERICA

## Abstract

Brain tumors, which are abnormal growths in the brain or central spinal canal, represent a serious global health threat. In 2023, they were responsible for 94,390 cases and 18,990 deaths in the US. Early detection with Magnetic Resonance Imaging is critical for effective treatment and higher survival rates. In this paper, we propose a method for the classification and localization of brain tumors in magnetic resonance imaging. Our method is based on the application of convolutional neural networks and for this task, we introduce and design a new network called the CBP_Net. The experimental analysis includes the Standard_CNN, a network designed by the authors, and other state-of-the-art networks, i.e., MobileNet, DenseNet, InceptionV3, ResNet50 and EfficientNetB0. This study emphasizes both quantitative and qualitative evaluations. The results demonstrate that the networks achieve optimal quantitative metrics: from 0.993 to 1.0 in accuracy. Qualitatively, we focus on visual explanations using Class Activation Mapping algorithms, specifically Grad-CAM and Score-CAM, to generate heatmaps highlighting relevant regions in the images. The Structural Similarity Index Measure is then employed to quantitatively measure the similarity between the heatmaps generated by the two CAM algorithms. This approach improves the reliability and trustworthiness of AI-driven brain tumor detection, helping clinicians diagnose.

## 1 Introduction

An abnormal growth of cells in the brain or central spinal canal is a brain tumor. There are more than one hundred distinct types of primary brain tumors, each with its own spectrum of symptoms, treatments, and outcomes. Brain tumors can be benign or malignant. The difference depends on the cell nature: non-cancerous and cancerous, respectively (https://www.aans.org/patients/conditions-treatments/brain-tumors). According to the Global Cancer Observatory in 2020, brain and central nervous system cancers represented an important proportion of global disease (1.9%) and 2.5% of deaths [1]. Furthermore, in 2023, there were around 94,390 new cases of brain cancer diagnosed in the US, and they correspond to 18,990 deaths with a relative survival rate of 35.7% (https://braintumor.org/brain-tumors/about-brain-tumors/brain-tumor-facts/).

Medical researchers are unsure of the primary causes and reasons behind why certain cells develop into tumor cells. It involves the person’s environment, genes, or both. Radiation, genetic predisposition, and other cancers spreading to other body organs are risk factors and possible causes of brain tumors. (https://www.hopkinsmedicine.org/health/conditions-and-diseases/brain-tumor).

When it comes to diagnosing brain tumors, magnetic resonance imaging (MRI) is the first diagnostic method that is used. It is a vital imaging tool for medical professionals because of its capacity to create finely detailed images of the soft tissues of the brain. MRI is a second-level, non-invasive procedure to obtain accurate images of the brain. It is a time-consuming device and also the cost of the MRI is remarkable, but the high-resolution guarantees and provides better soft-tissue contrast than other imaging modalities, such as CT scans, allowing for more thorough observation of brain structures and disorders. MRI assists in the investigation of the tumor location, size, shape, and level of involvement. It can also reveal information about the tumor type and grade based on its appearance and attributes. Moreover, MRI represents a larger family of advanced technologies that include functional MRI (fMRI), perfusion MRI, Magnetic Resonance Spectroscopy (MRS), and diffusion-weighted imaging (DWI), as well as the ability to use contrast agents. Treatment options vary depending on the kind, size, grade, and location of the brain tumor. Surgery, radiation therapy, radiosurgery, chemotherapy, and targeted therapy are all possible treatment options. In general, early detection guarantees a higher rate of survival and less invasive procedures [2]. Brain cancer is one of the most common cancers in the world, making detection, treatment, and prevention critical for public health and medical practice (https://www.cancer.org/cancer/types/brain-spinal-cord-tumors-adults/detection-diagnosis-staging/how-diagnosed.html).

Starting from these considerations, it is critical to use MRI for the early diagnosis of brain tumors, as this is crucial for the disease’s therapy. For the classification of brain tumors, Artificial Intelligence (AI), and specifically Deep Learning (DL), offers medical personnel effective assistance during the diagnosing process. Convolutional Neural Networks (CNNs) are a kind of Artificial Neural Networks (ANNs) and they represent specialized DL architectures designed for processing images, thanks to their ability to automatically learn hierarchical feature representations [3] Moreover, eXplainable AI (XAI) in healthcare, particularly biomedical imaging, has made progress in recent years. XAI can provide clinicians with important information, allowing them to make more accurate diagnoses during decision-making [4].

The contributions of this paper are below listed:

- we design and develop a method capable of distinguishing brain tumor images from MRI, applying CNNs.
- Starting from the five SOTA networks under consideration, i.e., MobileNet, DenseNet, InceptionV3, ResNet50 and EfficientNetB0, authors implement two other networks, such as the Standard_CNN, introduced in previous works [5,6], and the CBP_Net, a CNN designed for this classification task.
- The main contribution is the qualitative results section, thanks to the XAI algorithms. These algorithms are Class Activation Mapping (CAM), and in detail, the authors employ two CAM algorithms: Grad-CAM and Score-CAM.
- Finally, the authors perform a combination of qualitative aspects coming from the generated heatmaps and the quantitative measure of similarity, introducing the Structural Similarity Index Measure (SSIM). In this way, the two qualitative results (Grad-CAM and Score-CAM heatmaps) are quantified into a similarity value.

In the next section, the proposed method is presented, followed by the experimental analysis and results in Section 3 with a particular focus on subsection 3.1. In Section 4 the current state-of-the-art (STOA) is presented and a comparison with our approach. Lastly, the conclusion and future research goals are shown in the last section.

## 2 A method for explainable brain tumor detection

In this section, the steps of the method, from the dataset to the evaluation of the qualitative results are illustrated. Fig 1 shows the main phases.

**Fig 1:**
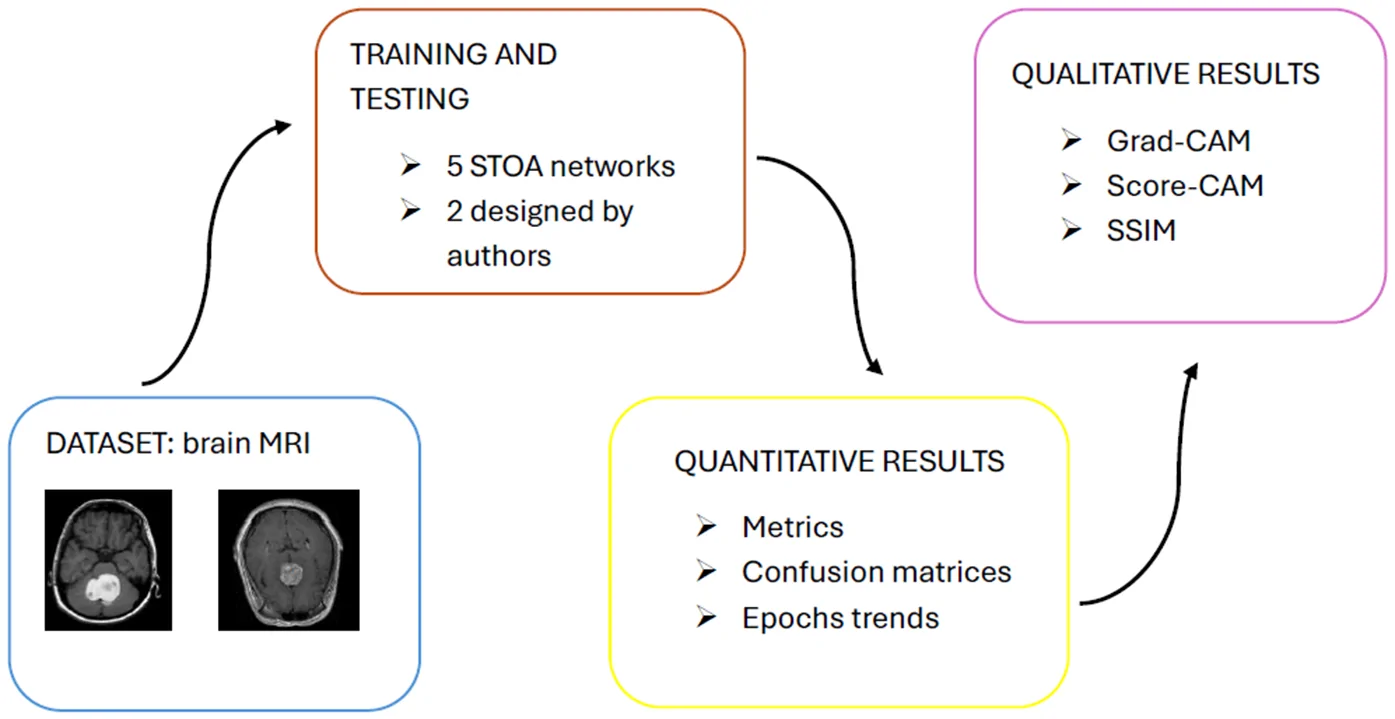
The workflow of the proposed method for brain tumor MRI classification and localization.

As shown in Fig 1, the first step regards the dataset. We decided to investigate MRI brain tumor detection using a public dataset divided into four classes: no_tumor, glioma, pituitary and meningioma. The technical details about the dataset are reported in Section 3. In Fig 2 shows several examples related to the four classes under analysis. The description of the disease (classes) is listed below:

- No_Tumor: a normal brain MRI image shows the brain’s various structures clearly, without any signs of abnormality. These structures include the cerebrum, cerebellum, brainstem, and ventricles.
- Glioma: Glial cells, the brain’s supporting cells, are the source of gliomas, a type of brain tumor. Accounting for about 30% of all cases, they are the most common type of primary brain tumor. Grade I gliomas are the least aggressive of the different types of gliomas, while grade IV gliomas (glioblastomas) are the most aggressive. The most popular imaging technique for glioma diagnosis is MRI. The grade of the tumor and other factors may affect how gliomas show up on MRI. Because gliomas contain a lot of fluid, they typically show up as bright on T2-weighted images in MRI studies, which are sensitive to fluid content. Gliomas augment with contrast, which means they absorb the contrast agent injected into the bloodstream. This can assist in identifying the tumor and determine its grade.
- Pituitary: The pituitary gland, which generates hormones including growth hormone, can develop abnormal growths called pituitary tumors. Whether or not pituitary tumors produce excess hormones determines whether they are classified as functional or non-functional. The sella turcica, a bony chamber at the base of the skull that contains the pituitary gland, is frequently the site of pituitary tumors on MRI. These tumors can range in size from tiny microadenomas (less than 1 cm) to massive macroadenomas (more than 1 cm). Nevertheless, some tumors have the ability to compress nearby tissues, including the optic chiasm, and expand beyond the sella turcica.
- Meningioma: Meningiomas develop from the meninges, the membranes that cover the brain and spinal cord. Meningiomas are usually harmless, but they might create issues if they become large enough to squeeze neighboring brain tissue or nerves. Meningiomas often exhibit a particular look on MRI, which is similar to well-circumscribed lobulated masses. They are typically isointense to gray matter on T1-weighted imaging and hyperintense on T2-weighted scans. Meningiomas frequently develop a “dural tail,” which is a linear or flame-shaped region of amplification that extends out from the primary tumor volume. This indicates the tumor’s connection to the dura mater.

**Fig 2:**
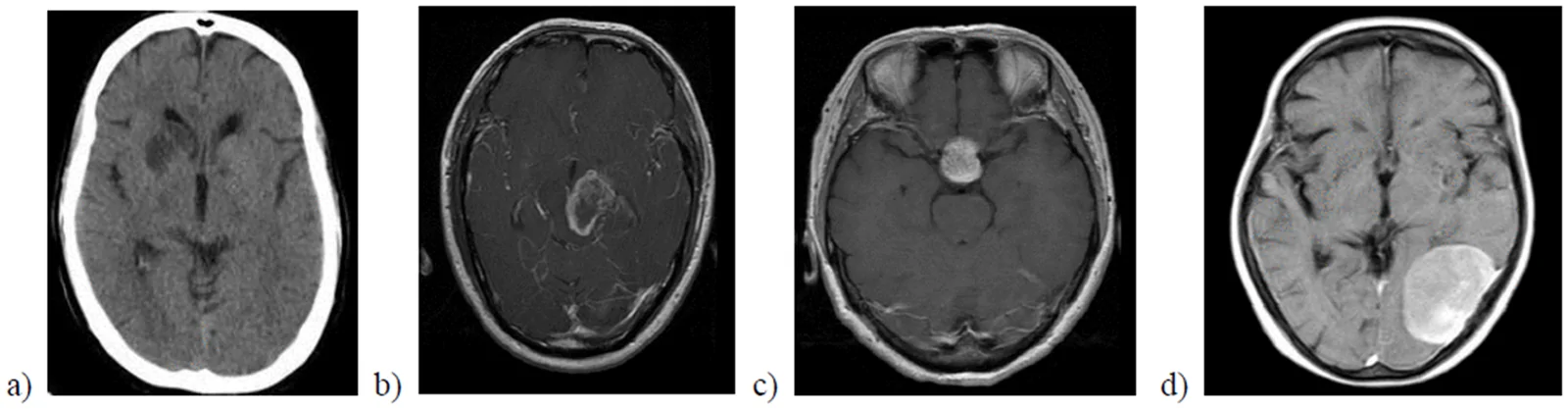
Brain MRI classes examples; a) No_tumor, b) glioma, c) pituitary, d) meningioma.

After the dataset was split, the models were trained, validated, and tested on seven CNNs; two of these, i.e., CBP_Net and the Standard_CNN, are developed by the authors. The CBP_Net is a network developed for brain tumor detection in MRI images, while the Standard_CNN was defined in other works [5,6]. Briefly, the latter is a combination of 14 layers. After the input layer, the architecture is composed of the alternation of the convolutional layer and MaxPooling. followed the Flatten layer and the combination of three dense layers (512, 256, 4) and the dropout layers with the rate of 0.5.

The CBP_Net model was specifically designed and optimized for this multi-class classification task. As shown in the code snippet in Listing 2, the network begins with a feature extraction stage composed of three sequential 2D convolutional layers using 32, 64, and 128 filters of size 3×3, each followed by a Rectified Linear Unit (ReLU) activation function and a 2×2 Max Pooling layer. Following a Flatten layer, the downstream classification (fully connected) pipeline consists of three Dense layers with 128, 64, and 4 neurons, respectively. To mitigate overfitting, a Dropout rate of 0.5 is applied immediately after the first two Dense layers. Finally, the output layer utilizes a Softmax activation function over the 4 target classes, and the model is compiled using the Categorical Cross-Entropy loss function and the Adam optimizer.

**Listing 1. The CBP_Net Python code snippet**

model = models.Sequential([

*# Feature Extraction Stage*

layers.Conv2D(32, (3, 3), activation='relu', input_shape=(self.input_width_height,

self.input_width_height, self.channels)),

layers.MaxPooling2D((2, 2)),

layers.Conv2D(64, (3, 3), activation='relu'),

layers.MaxPooling2D((2, 2)),

layers.Conv2D(128, (3, 3), activation='relu'),

layers.MaxPooling2D((2, 2)),

*# Classification Stage*

layers.Flatten(),

layers.Dense(128, activation='relu'),

layers.Dropout(0.5),

layers.Dense(64, activation='relu'),

layers.Dropout(0.5),

layers.Dense(self.num_classes, activation='softmax')

])

*# Model Compilation*

model.compile(

loss='categorical_crossentropy',

optimizer=Adam(learning_rate=self.learning_rate),

metrics=['acc', Precision(name="prec"), Recall(name="rec"), AUC(name='auc')]

)

In the follow we describe the remaining networks we considered:

- MobileNet [7,8]: network designed for embedded devices and mobile. This network prioritizes the efficiency and small model size, reducing the number of parameters. Moreover, this architecture guarantees a compromise between accuracy and speed through width and resolution multipliers.
- EfficientNetB0 [9,10]: this network has an architecture designed to find the best trade-off between performance and resource usage, so the main feature is the balance for scaling optimal performance. EfficientNet is available in a family of models (B0 through B7, as well as V2), with each growing in size and accuracy. The “B” number represents the scaling factor. Higher values indicate larger, more accurate models that need more processing resources.
- InceptionV3 [11,12]: this network focuses on efficient computation by using “Inception modules” that employ parallel convolutional filters of varying sizes. The network, with factorization techniques to reduce the number of parameters, emphasizes computational efficiency and diverse feature extraction. This architecture complexity provides a balance between accuracy and efficiency.
- ResNet50 [13]: network, also called Residual Network, combines residual connections to allow the network to learn identity mappings. This addresses the vanishing gradient problem, enabling the training of very deep networks. This type of ResNet consists in 50 layers, resulting deep achitecture, with high accuracy, but also high computational cost.
- DenseNet [14]:in this network, each layer is connected to every other layer, promoting feature reuse and information flow. This leads to more efficient learning and can reduce the number of parameters required. Strong feature propagation represents the main characteristic and difference compared to other networks.

Following training and testing, the CNNs are assessed for both quantitative and qualitative results. Metrics like accuracy, precision, recall, loss, and area under the curve (AUC) are computed for qualitative results.

Accuracy, which is determined by summing the true positives and negatives and dividing by the total number of studied samples, is the ratio of right predictions in a classification. The percentage of accurately predicted positive samples among all positive occurrences is known as precision. Recall is computed as the ratio of predictions designated to a specific class out of all observations that correspond to the class under examination. Loss is a measure of how well the model’s predictions match the observed values. Finally, AUC and in particular AUC-ROC (Area Under the Receiver Operating Characteristic Curve) assesses a model’s ability to differentiate between classes. It depicts the area under the ROC curve, which compares the true positive rate to the false positive rate at various thresholds (https://developers.google.com/machine-learning/crash-course/classification/roc-and-auc).

The quantitative results section includes the epochs-loss and epochs-accuracy trends for training and validation and the confusion matrix for testing. The latter are applied on the developed CBP_Net.

For the qualitative aspect, the next subsection explains the evaluation and the applied techniques in detail.

### 2.1 CAM application and explainability

This section explains the qualitative analysis and the applied algorithms. The qualitative analysis focuses on the CAM algorithms. These algorithms generate heatmaps in which common patterns in MRI images are highlighted, enhancing the explainability. In this way, a visual explanation of how a model classifies and which regions are relevant is performed. Moreover, CAM algorithms provide visual patterns regarding the regions that most influence the model’s predictions. However, in the absence of radiologist annotations or segmentation masks, these visual explanations indicate model attention rather than validated anatomical boundaries.

In this work, two CAM algorithms are considered to evaluate the models from a qualitative point of view: Gradient-weighted CAM (Grad-CAM) [15] and Score-weighted (Score-CAM) [16]. Grad-CAM employs the gradients of the target concepts flowing into the final convolutional layer to generate a heatmap. Essentially, it determines relevance based on the change in output compared to the feature maps and this determines the relevance of each feature map by taking the target class’s average gradient in relation to that feature map. However, gradients can sometimes be noisy or unreliable, leading to less accurate localization. Score-CAM, differently, avoids the usage of gradients. Instead, it is based on how the model’s prediction score changes as different regions of the feature maps are disrupted. Score-CAM, in fact, adopts a different method to describe weights and allows for the measurement of performance by scoring an extra metric known as Increase of Confidence.

In medical applications, DL models must be explainable in order to be considered for medical staff. Grad-CAM and Score-CAM contribute to this by providing visual explanations of the model’s decisions, which can increase trustworthiness and reliability among clinicians. The different CAM algorithms provide a more relevant explanation than a single CAM application. This phenomenon is caused by the fact that if more CAMs highlight the same pattern, the heatmaps will be more useful to medical staff because different functioning principles of the algorithms reveal the same region. This feature significantly enhances the model’s visual explainability and localization, supporting the final decision for a diagnosis.

In the final step of the method presented in Fig 1, the qualitative analysis focuses on the SSIM. This analysis is conducted on the heatmaps generated through the Grad-CAM and Score-CAM, applying a comparison sample by sample to measure the similarity in the images. This analysis is important because it measures the qualitative aspect, obtaining an output value between 0 and 1. Values close to zero represent images not similar to each other, while values close to one indicate that the two images are similar. Extending this concept to the brain tumor heatmaps coming from the CAMs, the optimal value is close to one because it means a high similarity between heatmaps generated through two different algorithms. This behavior enhances reliability and credibility, highlighting the common pattern in the images. Mathematically, SSIM relies significantly on statistical metrics such as mean, variance, and covariance to capture features in the images. The combination of brightness, contrast, and structural components is nonlinear, which helps SSIM better align with human perception. The SSIM between Grad-CAM and Score-CAM heatmaps was used specifically to quantify the inter-method consistency of the visual explanations across different architectural features, rather than to measure true anatomical localization accuracy. Since pixel-level expert annotations or segmentation masks were not available in the public dataset, SSIM serves as an indicator of explainability stability between gradient-based and perturbation-based methods. Further details on SSIM are available in [17].

## 3 Experimental analysis and results

This section reports the experimental analysis together with the qualitative and quantitative results and a discussion of the outcomes related to the proposed method.

We consider a freely available dataset from the Kaggle website (https://www.kaggle.com/datasets/masoudnickparvar/brain-tumor-mri-dataset?select=Training). The 7,023 brain MRI images in the dataset are categorized into four groups: meningioma, pituitary, glioma, and no tumor. The three datasets listed below are combined to create this dataset: SARTAJ dataset, figshare [18] (https://www.kaggle.com/datasets/sartajbhuvaji/brain-tumor-classification-mri) and Br35H (https://www.kaggle.com/datasets/ahmedhamada0/brain-tumor-detection?select=no). To guarantee a robust and unbiased evaluation of model performance and prevent potential data leakage, rigorous quality control protocols were applied to the dataset prior to model training. In the updated version of the dataset, explicit deduplication algorithms were considered across all candidate samples. Duplicate and near-duplicate images were systematically identified and eliminated, thereby ensuring that no overlapping or identical samples exist across the training, validation, and test subsets. The data were accessed for research purposes in January 2025. The authors had no access to information that could identify individual participants during or after data collection. All MRI images were provided in a completely de-identified and anonymous format by the source repositories. Furthermore, since the study relied exclusively on secondary analysis of publicly available, anonymized data, ethics committee approval was not required.

The dataset is split into training, validation, and testing folders in an 80-10-10 split and the number of images for each class is listed below:

- No_Tumor: 1,464 for training, 183 for validation, 184 for testing.
- Glioma: 1,119 for training, 139 for validation, 141 for testing.
- Pituitary: 1,017 for training, 127 for validation, 128 for testing.
- Meningioma: 1,146 for training, 143 for validation, 144 for testing.

All experiments were conducted on a workstation equipped with an Intel Core i7 CPU and 16 GB RAM. The software environment was built on Python 3.10.12 using TensorFlow 2.15.0 and Keras 2.15.0 frameworks on an Ubuntu 22.04 LTS operating system. We first investigated well-known network models such as ResNet50, InceptionV3, EfficientNetB0, DenseNet, and MobileNet. We trained and tested the Standard_CNN model, as well as the new CBP_Net. The choice of the hyperparameters is made after a series of testing and the final combinations are reported in the Table 1.

**Table 1:** Hyperparameters setting.

Epochs	Batch Size	Learning Rate	Image size
50	32	0.001	100x100x3

All input MRI images were preprocessed by resizing them to a uniform dimension of 100×100×3 pixels (RGB format) to match the required input shape for all evaluated architectures. All evaluated architectures were trained over 50 epochs exclusively on CPU infrastructure to assess their computational demand and training efficiency under resource-constrained conditions. Within this hardware setup, CBP_Net demonstrated the lowest computational footprint, completing its training phase in 17 minutes and 32 seconds, and achieving a total execution time of 18 minutes and 25 seconds for training and testing. In comparison, the other architectures required significantly higher computational timing; specifically, ResNet50, DenseNet and InceptionV3 required over 2 hours for training. This highlights that CBP_Net achieves a superior balance between computational efficiency and diagnostic performance, making it particularly suitable for deployment in hardware-limited clinical environments.

Regarding the transfer-learning training, pre-trained SOTA models (ResNet50, Inception V3, EfficientNetB0, DenseNet, and MobileNet) were loaded with weights pre-trained on the ImageNet dataset. A full fine-tuning approach was adopted: all convolutional backbones and classification layers were set as trainable (100% trainable layers) to allow the features to adapt completely to the medical imaging domain. Models were initialized using standard Keras/TensorFlow defaults. To ensure reproducibility, all random seeds were fixed to 42 across all training and evaluation runs.

The results obtained are summarized in Table 2.

**Table 2:** Metrics evaluation.

Network	Loss	Accuracy	Precision	Recall	AUC
**ResNet50**	$2.85\times 10^{-5}$	1.0	1.0	1.0	1.0
**InceptionV3**	0.451	0.998	0.998	0.998	0.999
**EfficientNetB0**	4.500	0.375	0.375	0.375	0.616
**DenseNet**	$2.0\times 10^{-4}$	1.0	1.0	1.0	1.0
**MobileNet**	0.015	0.998	0.998	0.998	0.998
**Standard_CNN**	0.045	0.993	0.993	0.993	0.993
**CBP_Net**	0.033	0.993	0.993	0.993	0.998

Examining Table 2, except for the EfficientNetB0, all the other network report optimal performance in terms of accuracy (0.98 to 1.0), precision and loss. However, these high values must be confirmed from the qualitative analysis performed in the next subsection. EfficientNetB0 underperformed significantly compared to other architectures, achieving an accuracy of only 37.5% and a high loss of 4.500. A detailed investigation revealed that this failure is attributable to the interaction between EfficientNet’s compound scaling strategy and the aggressive input downsampling to 100×100×3. EfficientNetB0 architectures are optimized for higher native input resolutions (e.g., 224×224 for B0); forcing a smaller input grid severely degraded spatial feature maps in deep layers, leading to gradient collapse during full fine-tuning without fine-tuned learning rate schedules or warm-up epochs.

The introduced networks, Standard_CNN and CBP_Net, follow the same behavior for the quantitative metrics. In the follow, we focus on these two networks, showing the confusion matrix in Fig 3 and the epoch-accuracy and epoch-loss trends reported in Fig 4.

**Fig 3:**
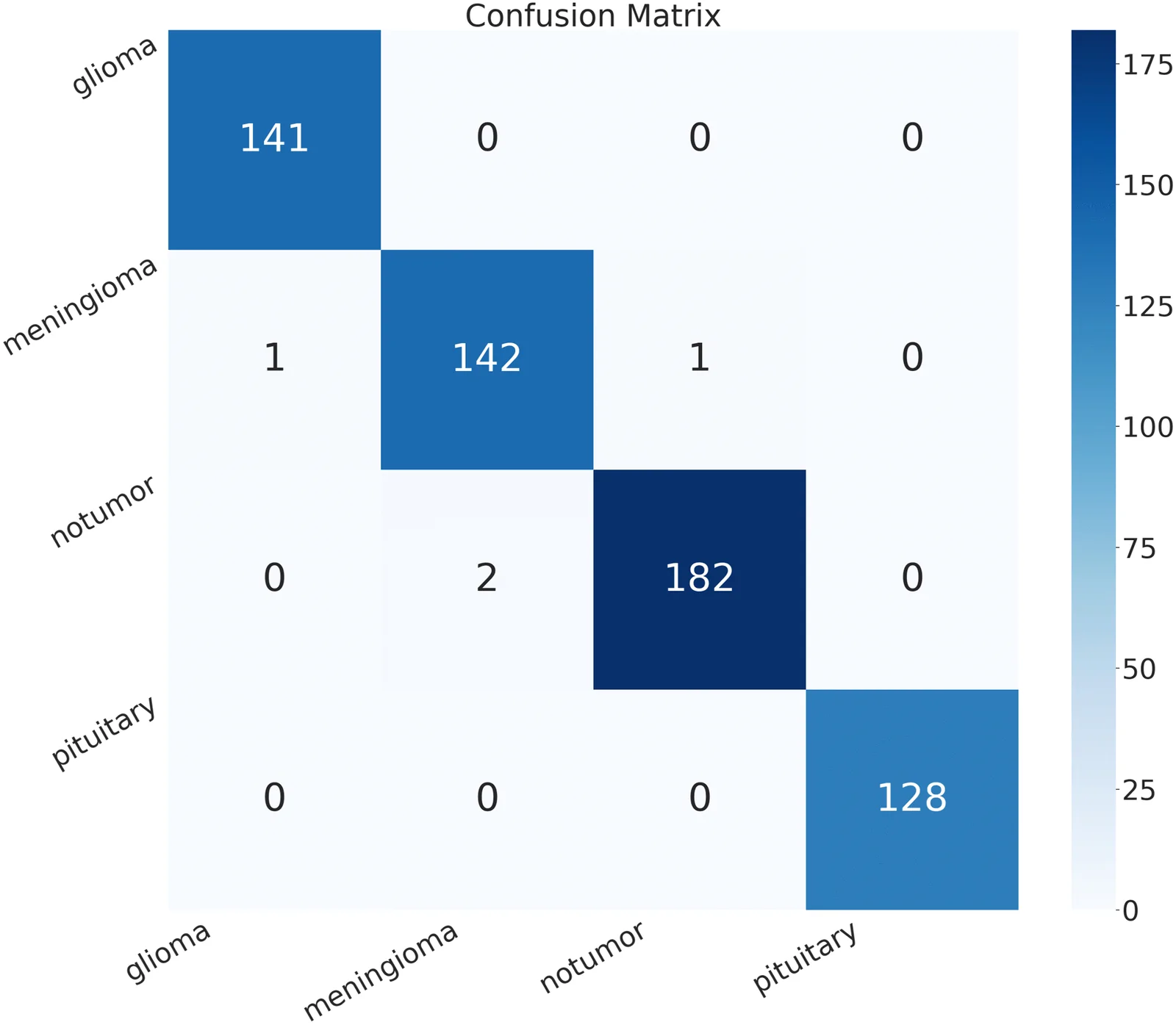
Confusion matrix for CBP_Net and Standard_CNN.

**Fig 4:**
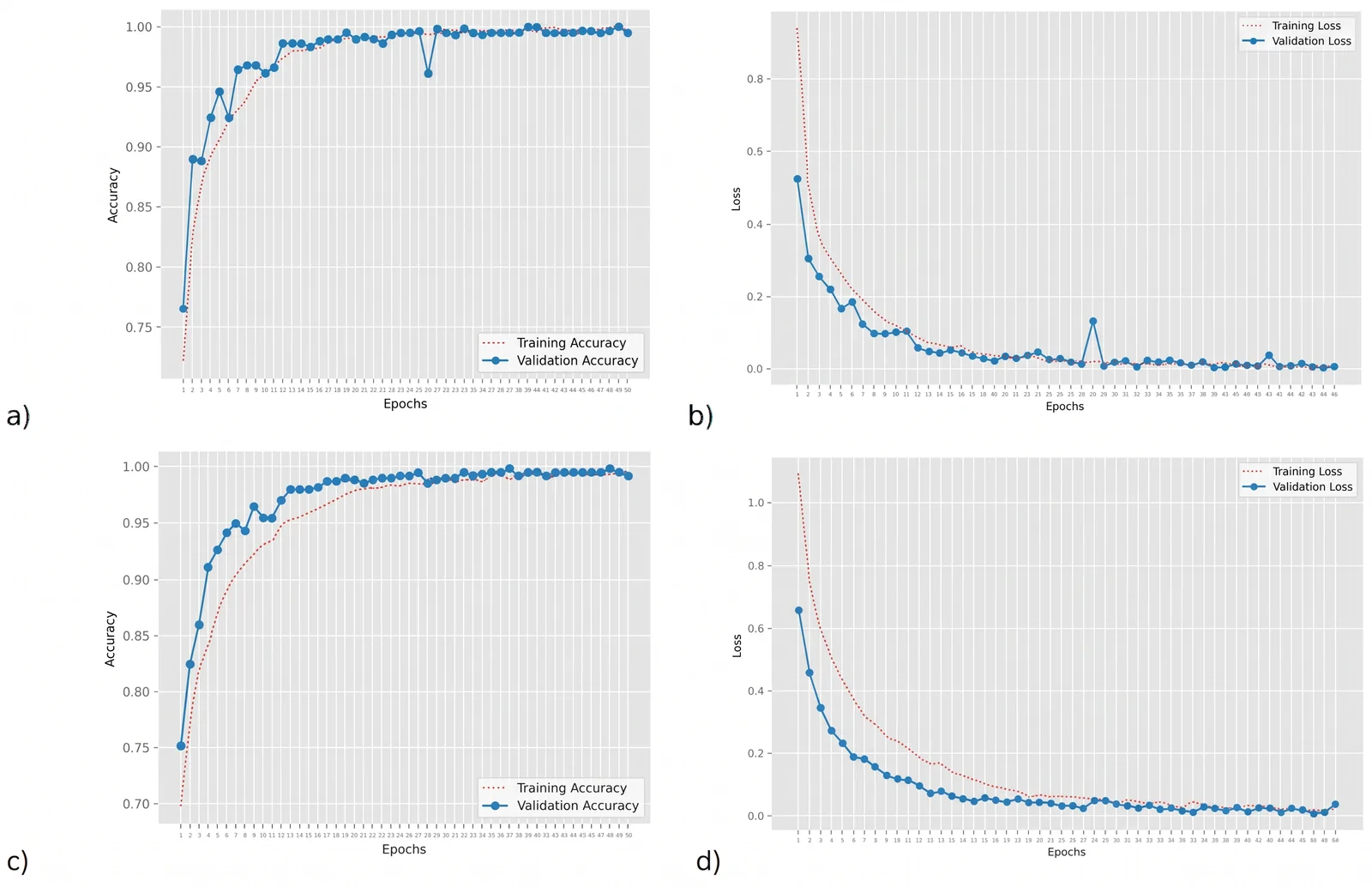
Plots trends; a) epoch-accuracy Standard_CNN, b) epoch-loss Standard_CNN, c) epoch-accuracy CBP_Net, d) epoch-loss CBP_Net.

Fig 3 represents the confusion matrix of the CBP_Net and the Standard_CNN. In the testing phase, the generated matrix for both models reported the same result, with high values on the first diagonal, corresponding to the correct prediction in the right class.

Fig 4 plots the trends over the epochs of accuracy and loss. As shown in the Fig 4, the dotted red line represents the training and the continuous blue line represents the validation phase. All four plots show optimal trends, avoiding the overfitting issues. This is because both the training and validation loss decrease consistently, and both the training and validation accuracy increase with a similar curve, excluding divergence in the trends. Fig 4c and 4d (CBP_Net) also report more stable trends with respect to Standard_CNN (Fig 4a and 4b).

### 3.1 Qualitative analysis

The qualitative results are reported and discussed in this section to provide real-world clinicians with useful visual decision-support cues by illustrating regions of interest prioritized by the model during brain tumor classification.

Fig 5 shows the heatmaps of the Grad-CAM application for the six networks, i.e., Densenet, InceptionV3, MobileNet, ResNet50, Standard_CNN and CBP_Net. Each figure comprises three images: on the left, the input brain MRI image; in the center, the generated heatmap; and on the right, the overlapping of the previous two.

**Fig 5:**
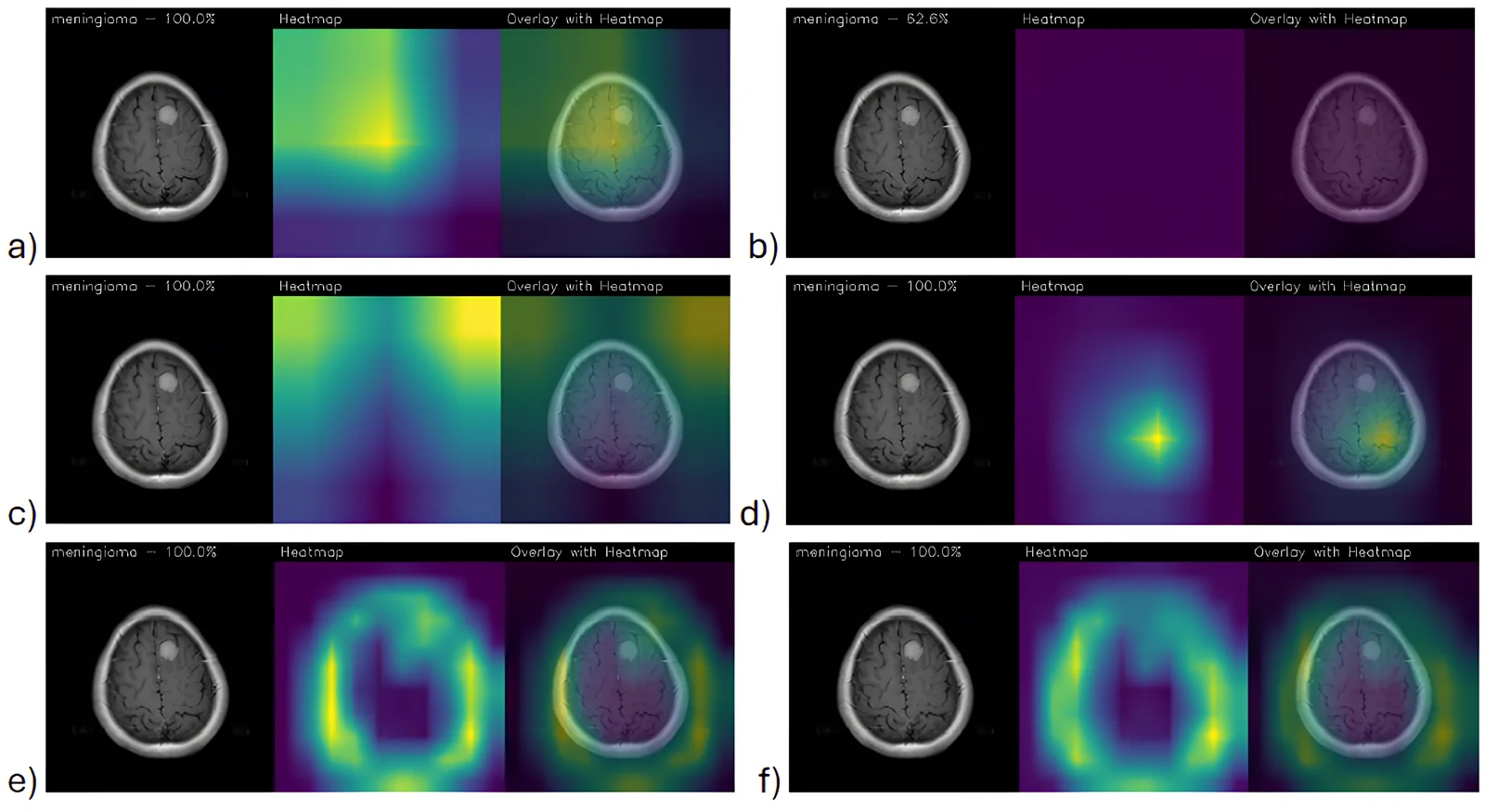
Grad-CAM; a) DenseNet, b) InceptionV3, c) MobileNet, d) ResNet50,e) Standard_CNN, f) CBP_Net.

All the heatmaps are generated on the same sample (meningioma class), with the tumoral mass present in the upper right of the brain. Fig 5a and 5c, which refer to the DenseNet and MobileNet heatmaps, respectively, highlight the upper external region of the brain failing to localize the diseases. A similar situation occurs for ResNet50 heatmaps (Fig 5d), but in this case, the Region Of Interest (ROI) is the lower right part of the brain, recognizing different patterns with respect to the useful one. InceptionV3 (Fig 5b) doesn’t activate the Grad-CAM, resulting in the worst possible result. On the other hand, the heatmaps of Standard_CNN and CBP_Net (Fig 5e and 5f) report the correct application of the Grad-CAM algorithm. The activated heatmaps are the border of the skull and the upper ROI in the right hemisphere, exactly the area with the meningioma. The two networks highlight the same areas with slight differences, enhancing the visual explanation of the output models.

The next step of the qualitative evaluation concerns the Score-CAM algorithm application. The Score-CAM heatmaps for Standard_CNN and CBP_Net network are shown in the Fig 6a and 6b. Also, the generated heatmaps highlight the skull and the tumor area with little difference in luminescence and shape of the regions. The Score-CAM of the other networks are not reported because they provide a similar situation of the Fig 5 (from a to d), with inactivation of the heatmaps, huge areas highlighted and no correspondence between the highlighted region and the disease’s localization. The Score-CAM confirms the good heatmaps and correct patterns to highlight for the clinicians.

**Fig 6:**
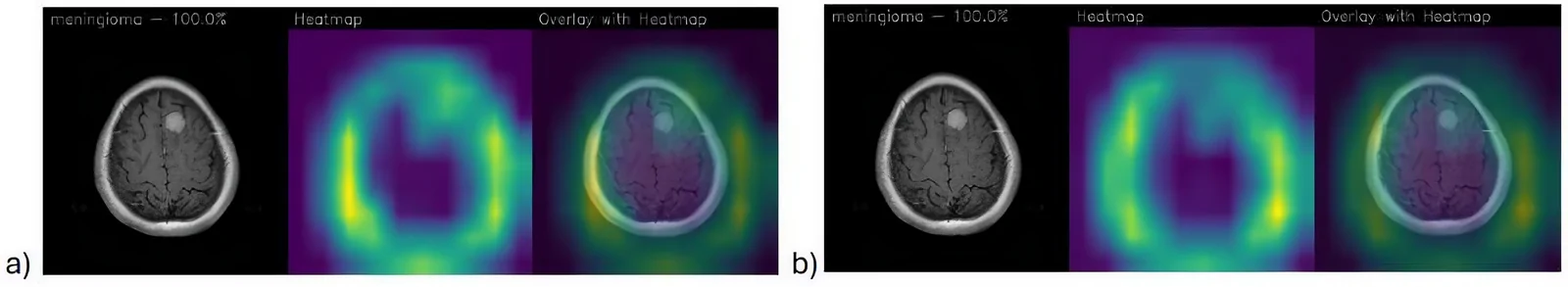
Score-CAM; a) Standard_CNN, b) CBP_Net.

The last phase of the qualitative aspect includes the application of the SSIM defined in the Section 2. Table 3 shows the SSIM values coming from the comparison sample by sample of the heatmaps of the Grad-CAM and Score-CAM for the CBP_Net and Standard_CNN.

**Table 3:** SSIM of the Grad-CAM/ Score-CAM comparison for the Standard_CNN and CBP_Net.

Networks	Classes	SSIM
Standard_CNN	no_tumor	0.965
	glioma	0.895
	meningioma	0.962
	pituitary	0.963
CBP_Net	no_tumor	0.974
	glioma	0.930
	meningioma	0.977
	pituitary	0.983

The table reveals good results of the output models of Standard_CNN and CBP_Net with high values in all the classes. These results indicate that the heatmaps of Grad-CAM and Score-CAM are similar, highlighting the same patterns but with slight variations concerning the different operating principles that are designed. Moreover, the SSIM represents a connection between the qualitative aspect, such as the areas of the heatmaps and the quantitative results, as they provide numerical values. So, in other words, the SSIM quantifies the qualitative results. CBP_Net reports values from 0.93 for the glioma class to 0.983 for the pituitary, while Standard_CNN exhibits values ranging from 0.895 for glioma to 0.965 for the no_tumor class. Higher values in all the classes in the CBP_Net provide a major similarity between the two sets of CAMs, making the CBP_Net output model more efficient in terms of pattern recognition with respect to the Standard_CNN.

At the end of our analysis, CBP_Net, which represents a variation of the Standard_CNN, is the choice for AI decision support in MRI. The network reports optimal metrics (0.993 in accuracy, precision and recall), with lower loss (0.033) with respect to the Standard_CNN (0.045), and correctly recognizes the common patterns in the images. In addition, CBP_Net provides higher values of SSIM in the four classes than Standard_CNN, guaranteeing a better AI-explanation and reliability.

## 4 Related work

In the following section, the authors present an overview of the current SOTA brain tumor recognition applying DL techniques. DL is employed for multiple applications [19], including cancer risk prediction [20], lesion classification [21,22] and segmentation, image reconstruction and generation [23,24]. A comprehensive survey by Wijethilake et al. [25] highlights several critical gaps in current glioma analysis, particularly emphasizing the’black-box’ nature of deep learning models and the lack of standardized metrics to validate the reliability of visual explanations for clinical use. While many studies achieve high performance, they often fail to address the consistency of the diagnostic regions identified.

In 2023, a review of Reham Kaifi [26] was published and this provides a complete overview of the main AI- techniques for brain tumor classification.

Starting with the work of Suitan et al. [27] which proposed a DL model, using their developed CNN composed by 16 layers, for brain tumor MRI classification. The model is tested on two datasets: one classifying tumors into meningioma, glioma, and pituitary, and the other differentiating between glioma grades (II, III, IV). Utilizing T1-weighted contrast-enhanced MRI images, the model achieves high accuracy, reaching 0.961 and 0.987 on the respective datasets, demonstrating its effectiveness for multi-class brain tumor classification. Respect to our method, no qualitative analysis was performed for he reliability.

In the study of Ye et al. [28], they enrolled 73 patients diagnosed with germinoma or glioma of the basal ganglia. Brain lesions were manually segmented based on both T1C and T2 FLAIR sequences. The T1C sequence was used to build the tumor classification model. A 2D convolutional architecture and transfer learning were implemented. ResNet18 from ImageNet was retrained on the MRI images of our cohort. Class activation mapping was applied for the model visualization. The model was trained using five-fold cross-validation, achieving a mean AUC of 0.88. By analyzing the class activation map, the model’s attention was focused on the peri-tumoral edema region of gliomas and tumor bulk for germinomas. This study showed that the T1C-based transfer learning model could accurately distinguish germinomas from gliomas of the basal ganglia preoperatively.In this similar task, our approach reports better metrics values.

Researchers in [29] designed a personalized CNN-based DL model for reliable glioma grade prediction and tested its performance utilizing transfer learning with AlexNet, GoogleNet, and SqueezeNet. The models were trained and evaluated using data from 104 clinical glioma patients (50 LGGs and 54 HGGs). The training data was increased using data augmentation techniques. A five-fold cross-validation approach was employed to evaluate each model’s performance. The custom model had accuracy, sensitivity, F1 score, specificity, and AUC values of 0.971, 0.980, 0.970, 0.963, and 0.989, respectively. No qualitative analysis was performed. In the paper of Solanki et al. [30], they explored automated brain tumor classification in medical images (MRI and CT scans) by comparing traditional ML and DL methods. They evaluated six classical ML classifiers, with Support vector Machine (SVM) achieving the highest accuracy. The study then moves to DL, employing CNNs and Deep CNNs, finding that VGG16 performs best among the DL models. Furthermore, they investigated various CNN architectures and dataset split ratios, concluding that a five-layered CNN with an 80:20 split ratio achieves a 0.9786 accuracy, demonstrating the superior performance of CNNs over traditional ML methods for this task. Also in this case no AI explanations was provide. Authors in [31] proposed a Bayesian Optimization-based method for automatically tuning the hyperparameters of CNNs) to improve brain tumor classification from MRI scans. The study utilized 3064 T1-weighted contrast-enhanced MRI images, classifying them into Glioma, Meningioma, and Pituitary tumors. The optimized CNN achieved a validation accuracy of 0.987, outperforming several pre-trained models (VGG16, VGG19, ResNet50, InceptionV3, and DenseNet201) and surpassing state-of-the-art methods on the same dataset. This demonstrated the effectiveness of automated hyperparameter tuning for enhancing CNN performance in brain tumor classification. Interesting work but without qualitative analysis.

Interesting work is the paper of Raza et al. [32] They proposed DeepTumorNet, a novel hybrid DL model for the accurate classification of brain tumors (BTs) from MRI images. The core contribution lies in modifying the GoogLeNet architecture by removing its final five layers and replacing them with fifteen new layers, alongside the implementation of a leaky ReLU activation function to enhance feature expressiveness. This modification addresses the limitations of traditional ML approaches, which rely on time-consuming hand-crafted feature extraction, and leverages the robust feature extraction capabilities of DL. DeepTumorNet was evaluated on a public dataset, achieving a remarkable 0.996 accuracy and precision, 1,0 recall, and a 0.9966 F1-score. These results demonstrated a significant improvement over state-of-the-art models, including AlexNet, ResNet50, and the original GoogLeNet, highlighting the effectiveness of the proposed architectural modifications for the classification of glioma, meningioma, and pituitary tumors.In this work, no qualitative analysis was included.

To address the common challenge of limited medical imaging datasets, recent studies have integrated generative models for data augmentation. Specifically, the TumorGANet framework, proposed by Nag et al. [33], combines transfer learning with Generative Adversarial Networks (GANs) to synthesize high-quality brain MRI images. By utilizing a GAN-based augmentation strategy alongside a fine-tuned ResNet50 architecture, this approach achieved a classification accuracy of 98.67%, a precision of 98.81%, and a recall of 98.67%. These results highlight the effectiveness of synthetic data in improving model generalization. While TumorGANet focuses on enhancing performance through data synthesis, our work achieves a higher accuracy of 99.3% with CBP_Net and prioritizes visual explainability by quantifying the reliability of diagnostic regions through the SSIM-based comparison of Grad-CAM and Score-CAM heatmaps.

Explainability has become a cornerstone in the development of reliable CAD systems. In this regard, Haque et al. [34] introduced NeuroNet19, an explainable deep neural network designed for the multi-class classification of brain tumors. By utilizing a modified CNN architecture on the widely used Figshare dataset, the authors achieved an accuracy of 99.11%, with high precision and recall values across glioma, meningioma, and pituitary tumor classes. Notably, NeuroNet19 incorporates Grad-CAM to visualize the decision-making process, ensuring that the model focuses on relevant pathological regions.

Moreover, the research of Zulfiqar et al. [35] focused on improving brain tumor classification using MRI images through a transfer learning approach with EfficientNetB0 models. This latter aims to accurately distinguish between glioma, meningioma, and pituitary tumors, crucial for early diagnosis. The study consisted of five EfficientNet variants (B0-B4) on a public dataset, demonstrating that EfficientNetB2 achieves the best performance with a 0.988 accuracy. The research also explored the impact of data augmentation and utilized Grad-CAM visualization to highlight tumor regions, showcasing the model’s effectiveness in identifying and classifying brain tumors. In our approach, Grad_CAM was integrated with the Score-CAM analysis and the SSIM, guaranteeing a better explanation.

An interesting approach is described in [36]. Authors proposed a DL-based method for brain tumor classification to improve the accuracy of computer-aided diagnostic systems (CADS) in IoT-healthcare. Addressing the issue of inaccuracy in existing AI-driven systems, the study utilizes pre-trained models ResNet-50, VGG-16, Inception V3, DenseNet201, Xception, and MobilleNet with data augmentation and transfer learning on brain MRI images. The proposed model demonstrated higher accuracy (0.985) compared to baseline models, suggesting its potential for reliable brain cancer diagnosis within IoT-healthcare systems. Further analysis for the explainability is not provided

Recent research has shifted towards more complex architectures and learning paradigms to enhance diagnostic precision. For instance, Dasanayaka et al. [37] explored the application of Vision Transformers (ViTs) for multi-class brain tumor classification, achieving a remarkable accuracy of 98.2% on a four-class dataset (glioma, meningioma, pituitary, and no tumor). Their work underscores the ability of attention mechanisms to capture global dependencies in MRI slices, which differs from the local feature extraction of standard CNNs. Parallelly, Samarasinghe et al. [38] addressed the challenges of data labeling by implementing a self-supervised learning (SSL) framework for tumor segmentation and edge detection. Using a combination of a pretext task (Jigsaw puzzle) and a downstream segmentation task, they reported a Dice coefficient of 0.89 and an IoU of 0.81, demonstrating that SSL can significantly improve the localization of tumor boundaries even with limited supervision.

Table 4 shows the cited related works with the comparison with our approach, considering the method applied, the results obtained, and if explainable AI techniques are included.

**Table 4:** State-of-the-art in brain tumor MRI classification.

Authors	Method	Result	Explainbility
Suitan et al. (2019)	Their 16-layer CNN	0.961 accuracy 0.987 accuracy	no
Ye et al. (2022)	ResNet18	0.88 AUC	yes
Ozcan et al. (2021)	AlexNet, GoogleNet,SqueezeNet	0.971 accuracy	no
Solanki et al. (2023)	SVM and VGG16	0.9786 accuracy	no
AIt et al. (2022)	VGG16, VGG19, ResNet50, InceptionV3, and DenseNet201	0.987 accuracy	no
Raza et al. (2022)	DeepTumorNet	0.996 accuracy	no
Nag et al. (2022)	TumorGANNet	0.986 accuracy	no
Haque et al. (2024)	CAD system/ NeuroNet19	0.991 accuracy	yes
Zulfiqar et al. (2023)	5-variant of EfficientNet (b0-B4)	0.988 accuracy	yes
Haq et al. (2022)	ResNet-50, VGG-16, Inception V3, DenseNet201, Xception, and MobilleNet	0.985 accuracy	no
Dasanayaka et al. (2022)	Vision Transformers	0.982 accuracy	no
Samarasinghe et al. (2025)	Self-supervised learning	0.89 dice coefficient	no
Our work	MobileNet, EfficientNetB0, ResNet50, InceptionV3, DenseNet, Standard_CNN CBP_Net	0.993 accuracy	yes

## 5 Conclusion and future works

Brain tumors represent one of the most dangerous diseases and their early detection and classification are crucial to patient health. In this paper, authors proposed a method to classify a four-class dataset, introducing the CBP_Net. The quantitative and qualitative evaluation revealed how SOTA networks, such as DenseNet and MobileNet, reach good values of accuracy, precision and loss; however, the generated heatmaps showed their limitations, not focusing on the relevant areas of the MRI brain images. On the other hand, Standard_CNN and even more the CBP_Net guaranteed an optimal visual explanation, Standard_CNN and particularly CBP_Net demonstrated high inter-method consistency between Grad-CAM and Score-CAM heatmaps, as quantified by high SSIM values, indicating stable visual attention patterns across different explainability frameworks. This behavior was reinforced by the application of two CAM algorithms: Grad-CAM and Score-CAM and their combination into the SSIM, with high values for the CBP_Net. It is important to observe that the current evaluation is limited by the absence of cross-validation, confidence intervals, and testing on independent external datasets.

Future work will incorporate repeated cross-validation experiments and multi-center validation to further strengthen the statistical robustness and clinical generalizability of the proposed approach. The CPB_Net will be applied for the brain tumor recognition with more kinds of brain tumors, enlarging the multi-class task. As a primary direction for future work, we plan to validate the generated heatmaps against expert radiologist annotations and segmentation masks using quantitative localization metrics (such as IoU and Dice Similarity Coefficient) to rigorously evaluate the clinical localization reliability of the proposed approach.
